# Vessel pruning or healing: endothelial metabolism as a novel target?

**DOI:** 10.1080/14728222.2017.1282465

**Published:** 2017-01-27

**Authors:** Anna Rita Cantelmo, Andreas Pircher, Joanna Kalucka, Peter Carmeliet

**Affiliations:** ^a^ Laboratory of Angiogenesis and Vascular Metabolism, Department of Oncology, KU Leuven, Leuven, Belgium; ^b^ Laboratory of Angiogenesis and Vascular Metabolism, Vesalius Research Center, Center for Cancer Biology (CCB), VIB, Leuven, Belgium

**Keywords:** Angiogenesis, tumor vessel normalization, endothelial cell metabolism, anti angiogenic therapy

## Abstract

**Introduction:** Antiangiogenic drugs were originally designed to starve tumors by cutting off their vascular supply. Unfortunately, when these agents are used as monotherapy or in combination with chemotherapy, they provide only modest survival benefits in the order of weeks to months in most cancer patients. Strategies normalizing the disorganized tumor vasculature offer the potential to increase tumor perfusion and oxygenation, and to improve the efficacy of radio-, chemo- and immunotherapy, while reducing metastasis.

**Areas covered:** This review discusses tumor vascular normalization (TVN) as an alternative strategy for anti-angiogenic cancer treatment. We summarize (pre)-clinical strategies that have been developed to normalize tumor vessels as well as their potential to enhance standard therapy. Notably, we describe how targeting endothelial cell metabolism offers new possibilities for antiangiogenic therapy through evoking TVN.

**Expert opinion:** Several drugs targeting VEGF signaling are now clinically used for antiangiogenic cancer treatment. However, excessive blood vessel pruning impedes perfusion and causes tumor hypoxia, known to promote cancer cell dissemination and impair radio-, chemo- and immunotherapy. Normalized vessels lessen tumor hypoxia, impair cancer cell intravasation and enhance anticancer treatment. New data indicate that targeting endothelial cell metabolism is an alternative strategy of antiangiogenic cancer treatment via promotion of TVN.

## 1. Introduction

Angiogenesis is the process of new blood vessel formation [1]. It occurs throughout life in both health and disease and relies on migration, proliferation, and differentiation of endothelial cells (ECs), which line the inside wall of blood vessels. In established vessels, ECs have a cobblestone-like appearance and are often referred to as phalanx cells [2]. However, in the presence of pro-angiogenic signals such as growth factors and hypoxia, ECs can rapidly switch to an angiogenic state and become motile and invasive [3,4]. Upon detection of pro-angiogenic stimuli, such as VEGF (vascular endothelial growth factor), ECs lose their adherence junctions, and matrix metalloproteases degrade the basement membrane, thereby creating a scaffold for EC migration [3,4]. In response to such stimuli, a vessel sprout emerges in which individual ECs adopt distinct and functionally specialized phenotypes [3].

Vessel sprouting is initiated by the differentiation of ECs into specialized tip and stalk cells. Tip cells extend long filopodia and guide the new sprout toward an angiogenic stimulus but proliferate rarely, whereas stalk cells have fewer filopodia, proliferate to elongate the branch and form a vascular lumen [3,4]. When the new branch is formed and perfused, ECs regain their quiescent phalanx cell phenotype. Recruitment of pericytes and vascular smooth muscle cells, together referred to as mural cells, provides stability, maintains vessel integrity and regulates perfusion [3,4]. Furthermore, extracellular matrix proteins, laid down by both phalanx cells and pericytes, establish a basement membrane at the basal side of the endothelium [3–5].

Blood vessels not only deliver oxygen and nutrients to the body’s tissues but also support diseases such as cancer [6]. Rapidly growing tumors are in continuous demand for oxygen and nutrients. Therefore, they have an excessive production of angiogenic stimuli, which creates an imbalance in pro- versus antiangiogenic signaling. This results in an abnormal, leaky, and hypoperfused vascular network, characterized by hypoxia, acidosis, and high interstitial fluid pressure. This hostile tumor microenvironment stimulates the production of pro-angiogenic factors even more and fuels an endless self-reinforcing loop of nonproductive angiogenesis [7,8]. Paradoxically, however, this nonproductive angiogenesis leads to the formation of less functional vessels. This results in hypoxic sites within the tumor, deprived of nutrients and growth factors, creating a hostile microenvironment, from where cancer cells attempt to escape, thereby favoring cancer cell invasion and dissemination, further aided by the leaky endothelium, through which cancer cells can escape [7,8]. Moreover, the abnormal tumor vasculature impairs perfusion and thus drug delivery and distribution. Together, these features stimulate overall malignancy [7,9,10].

Therapeutic approaches for inhibition of angiogenesis have been developed to treat cancer and have led to the approval of several antiangiogenic drugs. So far, innumerable patients have benefited from these therapies but limited efficacy and resistance pose clinical challenges [8,11–13]. A novel paradigm is to heal the abnormal tumor vasculature in a process called tumor vessel normalization [10]. Tumor vascular normalization (TVN) strategies reduce metastasis and improve the response to conventional anticancer therapies [10,14].

## 2. Role of abnormal vasculature in tumor progression and therapeutic strategies

### 2.1. Tumor vessels are highly abnormal

**Figure 1.**
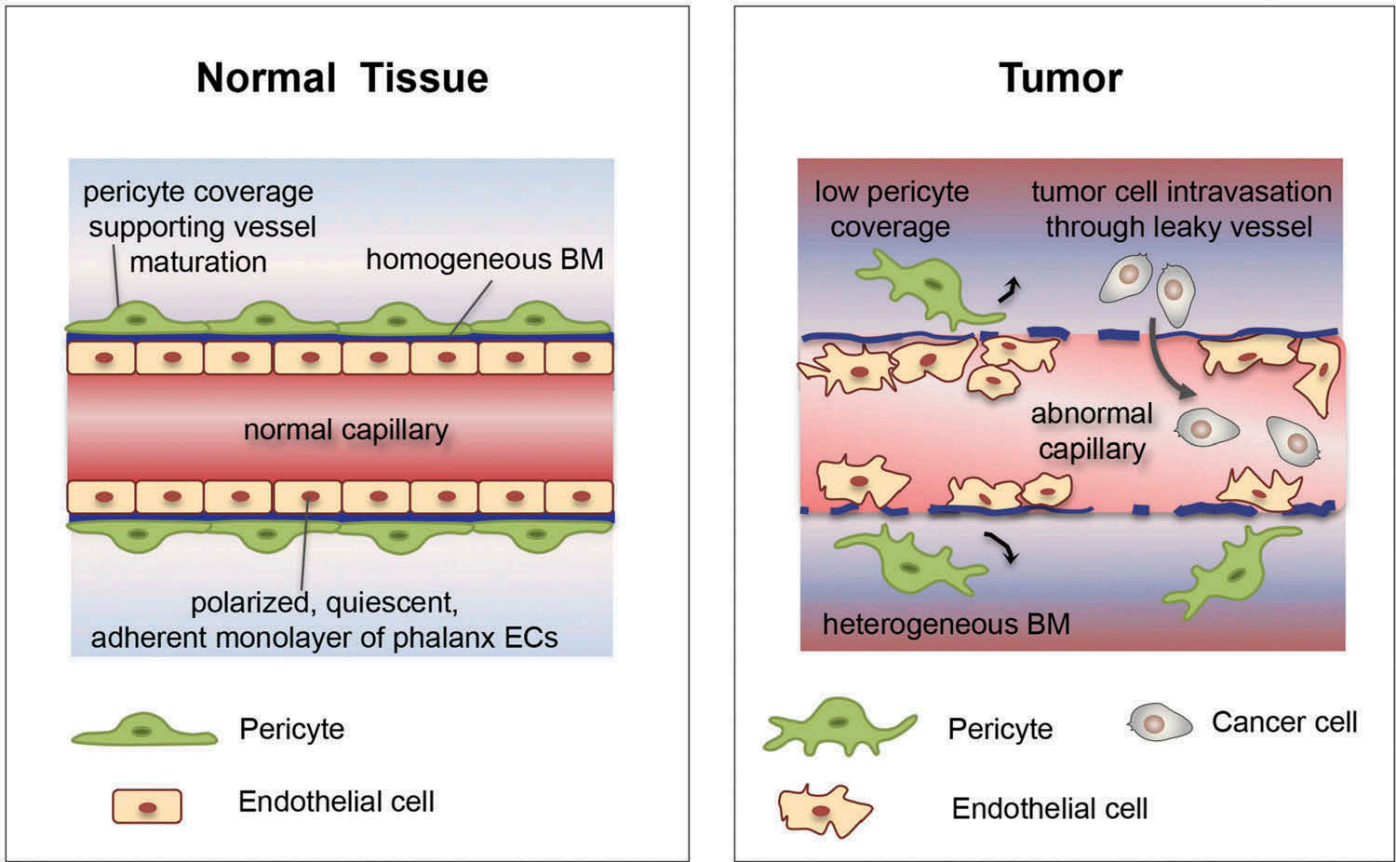
Tumor vessels are structurally and functionally abnormal. Endothelial cells lining tumor vessels demonstrate aberrations in shape, they are hyperproliferative and hypermigrative and are often separated by wide and irregular inter-endothelial junctions. In addition, tumor vessels are covered by fewer pericytes, which are often detached from endothelial cells. These structural abnormalities lead to hypoperfusion and hypoxia, which stimulate cancer cells to escape and metastasize in distant organs. Moreover, the accompanying functional aberrations limit delivery and distribution of chemotherapeutics to and into the tumors. BM: basement membrane. Adapted from [9].

Tumor blood vessels are structurally and functionally highly abnormal. They are tortuous, leaky, irregular and form a chaotic network. Moreover, they are heterogeneous in size and shape, ranging from capillaries to big, thin-walled vessels [7,9,10] (Figure 1). ECs lining tumor vessels have an irregular shape and are disorganized. They establish weak junctions, which results in a loose association, promoting EC trans-migration from their resident site. In certain regions, ECs are stacked upon each other and extend multiple protrusions within the lumen, thus obstructing the blood flow, while at other sites, ECs move away from their position, leaving gaps behind. In addition, mural cells show abnormal structural features. They have an abnormal shape and are often loosely associated with ECs or absent. Finally, the basement membrane of tumor vessels is also abnormal. In some tumors, it is unusually tick, while very thin, discontinuous, or absent in others [7,9,10].

Abnormalities in tumor vessel morphology impair perfusion. Blood flow often changes direction, flowing rapidly in certain vessels and being stagnant in others. In addition, the structural abnormalities lead to increased vessel permeability. Hence, intravascular fluids and plasma proteins can easily extravasate and increase interstitial fluid pressure, further impeding blood flow. Moreover, these leaky vessels facilitate intravasation of cancer cells and metastasis [8–10]. The combination of poor perfusion and increased interstitial fluid pressure creates hypoxic and acidic sites. In such conditions, invasive cancer cells are selected. Overall, the aforementioned tumor vessel abnormalities increase metastasis and impair the delivery and efficacy of systemically administered drugs, therefore reducing the efficacy of anticancer therapies [7–10].

### 2.2. Vascular normalization as anticancer strategy: an emerging paradigm

Anti-VEGF therapies were originally designed to restore the balance between pro- and antiangiogenic molecules and the vascular architecture of tumors by pruning immature vessels [15–17]. Early preclinical studies were promising and demonstrated a significant tumor growth delay and reduced metastasis [16,18]. Unfortunately, the effects of such anti-VEGF agents in cancer patients during clinical trials have not fulfilled the expected hopes as the survival improvement by antiangiogenic therapy is rather modest [19]. Monotherapy with anti-VEGF monoclonal antibody often failed to attain substantial response rates or survival benefits [20–22]. These data suggest that anti-VEGF therapy alone cannot efficiently induce sustained tumor shrinkage or complete tumor eradication in most cancer patients.

However, anti-VEGF therapy in combination with systemic chemotherapy has often shown improvements in progression-free survival when compared with chemotherapy [21,23–28]. This has been interpreted to imply that anti-VEGF therapy may enhance the efficacy of systemic chemotherapy. These findings are counterintuitive since anti-VEGF therapies were designed to promote vascular pruning; yet, the efficacy of chemotherapy relies on adequate tumor blood supply to ensure drug delivery. Thus, tumor vessel pruning by anti-VEGF treatment should theoretically decrease rather than enhance the chemotherapy efficacy [10]. The hypothesis of vascular normalization may resolve this paradox [29]. This hypothesis posits that rather than destroying vessels, antiangiogenic therapy might restore the normal structure and function of tumor vessels by ‘healing’ abnormal disorganized and dysfunctional tumor vessels. These changes would improve tumor oxygenation, thereby reducing metastasis and improving therapy responses, in part through more efficient drug delivery and lessened tumor hypoxia (which can improve the efficacy of several chemo- or radiotherapeutics, given that they rely on the conversion of oxygen to its radicals, and of immunotherapy [see below]). Thus, anticancer therapies given upon normalization might achieve greater efficacy [29].

Initial evidence for vascular normalization stems from preclinical studies with anti-VEGF, which show that blockade of VEGF signaling remodels the abnormal tumor vasculature into a more normal vasculature [14]. Of relevance, the kinetics of vascular normalization determined the overall outcome of combined antiangiogenic and conventional therapy. Preclinical and clinical tumor studies with anti-VEGF agents identified a ‘normalization window,’ typically occurring within a few days after therapy onset followed by a closure coinciding with the loss of normalization features [10]. The transient nature of the normalization of tumor vessels may relate either to excessively high or continued dosing of antiangiogenic therapy or to the development of resistance by activation of alternative pro-angiogenic pathways [10]. High doses of antiangiogenic drugs prune the immature tumor vessels, causing a rapid reduction in blood perfusion and consequent increase in hypoxia, which is a strong stimulus for cancer cells to invade, intravasate, and metastasize [8]. On the contrary, properly timed low dose of anti-VEGF therapy promotes the formation of less tortuous vessels, normal basement membrane, and greater vessel coverage by pericytes. Indeed, low doses of anti-VEGFR2 antibody normalized the breast cancer vasculature and improved tissue perfusion, reprogramming the tumor microenvironment from immunosuppression toward enhanced cancer vaccine therapy [30].

Other mechanisms might contribute to the vascular normalization effect of anti-VEGF agents. For instance, upon anti-VEGFR2 inhibition, mural cells upregulate angiopoietin-1 (ANG-1), promoting vessel maturation, stability, and integrity [31]. The ANG/TIE-2 system represents another target for antiangiogenic therapies to induce tumor vessel normalization [1,8,10]. In contrast to ANG-1, ANG-2 is primarily synthesized by ECs and antagonizes the effects of ANG-1. ANG-2 destabilizes blood vessels and promotes vascular permeability. Hence, preclinical reports demonstrated that blockade of ANG-2 induces vessel normalization by recruitment of pericytes and tightening of endothelial junctions [32,33]. Moreover, combined targeting of VEGF and ANG-2 showed a greater degree of vessel normalization [34].

### 2.3. Emerging successful tumor vessel normalization strategies

In addition to targeting the VEGF/VEGFR and ANG/TIE-2 axes, a number of strategies that alter other molecules in stromal and cancer cells can induce vascular normalization. The oxygen sensor prolyl hydroxylase domain protein 2 (PHD2) plays an important role in mediating abnormalities in tumor vessels. In the tumor microenvironment, lack of oxygen reduces the enzymatic activity of PHD2 on one hand, and possibly as a feedback, also upregulates the expression levels of this enzyme. Nonetheless, hypoxia results in the activation of hypoxia-inducible transcription factors, key mediators of transcriptional response to hypoxia [35]. In turn, VEGF is upregulated, thereby promoting abnormal angiogenesis [36]. Studies in genetically modified mice have shown that global (stromal) or endothelial-specific PHD2 haplodeficiency does not impair physiological angiogenesis but induces sustained normalization of tumor vessels [2,37]. There are currently no pharmacological agents available to inhibit PHD2 specifically in ECs. In addition, global PHD2 haplodeficiency also induces tumor vessel normalization in part by decreasing the activation and contraction of cancer-associated fibroblasts (CAFs) [38]. Mechanistically, PHD2 haplodeficient cancer cells inhibit CAF-induced matrix deposition that cancer cells use as a migration scaffold for dissemination, thereby reducing metastasis [38]. This suggests that administration of a pharmacological blocker inhibiting PHD2 in both stromal and cancer cell compartments might offer therapeutic benefit by reducing metastatic disease.

Specific targeting of the transmembrane glycoprotein L1 on tumor vasculature also promotes tumor vessel normalization, resulting in reduced tumor growth and metastasis [39]. The regulator of G-protein signaling 5 (RGS5) is a marker of mural cells that has been identified as a key mediator of the abnormal tumor vasculature [40]. Tumor vessels of RGS5-deficient mice were characterized by reduced vessel permeability and leakage, increased structural homogeneity, improved oxygenation, and coverage by more mature pericytes. This enhanced the influx of immune effector cells into the tumor and prolonged the survival of the tumor-bearing mice [40], offering alternative therapeutic opportunities for immunotherapy and anticancer therapy [40]. The modulation of tumor-associated macrophages (TAMs) may be another way to control tumor vessel abnormalities. For instance, histidine-rich glycoprotein suppressed the expression of placental growth factor and induced the polarization of TAMs, promoting TVN and enhancement of immunity [41].

The anti-malaric drug chloroquine also yielded promising results in cancer patients [42]. While its anticancer cell activity relies on the blockade of cancer cell autophagy at a high dose, a low dose of chloroquine targets ECs via an autophagy-independent increase in Notch signaling, which promotes tumor EC quiescence and thereby tumor vessel normalization [43,44]. A number of FDA-approved drugs also target molecules that indirectly contribute to the tumor vessel abnormalities, namely by targeting cancer cells that subsequently affect tumor vessels. Examples include the human epidermal growth factor receptor 2, the phosphoinositide-3-kinase/AKT serine/threonine kinase 1/mammalian target of rapamycin (mTOR) axis, Ras, and the EGF receptor (reviewed in Ref. [10]).

Taken together, the aforementioned studies suggest that targeting stromal, cancer, and other cells in the tumor milieu may improve the efficacy of tumor vessel normalization. The challenge for the future is to explore whether these approaches also promote sustained tumor vessel normalization in patients in clinical settings, as observed in preclinical studies.

### 2.4. Vessel normalization as strategy to improve cancer immunotherapy

In addition to the examples mentioned above, a growing body of evidence suggests that tumor vessel normalization may also enhance the efficacy of immunotherapy [30,40,41,45]. During tumor progression, cancer cells co-opt immune checkpoint pathways to promote immune evasion, particularly by cytotoxic T cells [46]. Different strategies to interfere with ligand–receptor interactions involved in immune checkpoint pathways have been developed and have entered clinical practice [47]. However, several hurdles need to be overcome to further improve such treatments.

Indeed, the efficacy of anticancer immunotherapy by blocking immune checkpoints is hampered by hypoxia and poor infiltration of T cells inside the tumor, as a result of the poor perfusion in the disorganized tumor vessels [48]. Abnormal tumor vessels also limit the adhesion and extravasation of leukocytes and impair their infiltration inside the tumor core [7,9]. Hypoxia increases the immunosuppressive nature of the stromal tumor environment, by impairing T-cell effector functions (T-cell receptor signaling, proliferation, and cytokine production by T cells) [48,49]. In contrast, hyperoxia increases cytotoxic T-cell performance, which correlates with better clinical responses to blockade of the immune checkpoint molecule programed death 1 (PD-1) [50]. In addition, nutrient deprivation (such as glucose) impedes T-cell proliferation and activation into CD8^+^ effector cells [51].

Hence, tumor vessel normalization increases tumor perfusion, and thereby oxygen and nutrient supply, and thus can be expected to improve the overall anticancer immunotherapy response. This hypothesis is supported by findings that anti-VEGF therapy promotes antitumor immunity, in part by improving vessel function and reducing hypoxia [30]. Restoring vessel integrity improves tumor perfusion and decreases interstitial fluid pressure, processes that would be expected to improve the influx of immune cells into the tumor [40,52]. Moreover, the fewer gaps between ECs in the normalized vasculature might create a more continuous vascular surface to support leucocyte rolling and diapedesis [53]. However, the exact mechanism by which tumor vessel normalization strategies increase the influx of immune cells into the tumor, while reducing cancer cell escape, remains to be defined. The increased antitumor immunity responses observed upon anti-VEGF therapy might be also related to the inhibition of the immunosuppressive functions exerted by VEGF on effector T cells [54]. Thus, blocking VEGF signaling enhances effector T-cell function by increasing their activation and delivery to the tumor (via tumor vessel normalization) on one hand and by inhibiting the VEGF-induced upregulation of inhibitory immune checkpoints, on the other hand [30,55].

### 2.5. Vessel normalization: a clinical perspective

Unfortunately, the high expectations of antiangiogenic therapies could not be fulfilled, as the high therapy efficacy observed in preclinical models could not be fully reproduced in clinical trials [56]. Therapy effects in cancer patients are mostly short lived due to intrinsic refractoriness or development of acquired resistance upon antiangiogenic therapy. Several modes of resistance have been identified preclinically, but they are less well characterized in the clinical setting [57,58].

Limitations to better clinically characterize the mode of action of antiangiogenic therapies are related to multiple reasons. Amongst those, one reason may relate to the fact that most antiangiogenic drugs are used for the treatment of advanced stage disease (mostly metastatic), where tissue sampling is only possible from accessible tumors (for instance, rectal cancer via rectoscopy); and therefore, translational investigations are often limited to blood sampling or imaging studies [19]. Nevertheless, in a subset of patients, tumor vessel normalization has been observed upon administration of antiangiogenic therapy [59] (Table 1).

**Table 1.** Clinical studies investigating vascular normalization in humans.

Tumor type	Therapeutic strategy	Measurement of vascular normalization	Clinical finding and translational observation
Rectal cancer [60]	Bev in combination with radio-chemotherapy	Tissue biopsies, tumor imaging by FDG-PET and functional dynamic CT	TVN upon bev monotherapy (radiological and histological evaluation), local control rate and DFS in phase II study
Glioblastoma multiforme [61]	Cediranib	Tumor perfusion by DCE-MRI	Perfusion response (increase) correlated with prolonged OS
NSCLC [62]	Bev in combination with doublet chemotherapy	Tumor perfusion by perfusion CT (MTT)	Prolongation of MTT correlated with prolonged OS
NSCLC [63]	Bev in combination with doublet chemotherapy	Tumor uptake of radiolabeled chemotherapy	Reduced chemo uptake after bev application
Breast cancer [64]	Bev with combinational chemotherapy	Tissue biopsies after bev monotherapy (single dose)	Predictive pre-therapy MVD
Breast cancer [65]	Sunitinib with chemotherapy	Tissue biopsies, DCE-MRI	Increased VNI and perfusion in the combinational treatment arm
Colorectal cancer [66]	Bev in combination with 5-FU	^18^F-5-FU PET/CT scanning	Reduced 5-FU trace uptake short term after bev application

bev: bevacizumab; FDG-PET: 18-fluorodeoxyglucose positron emission tomography; CT: computed tomography; MVD: microvessel density; NSCLC: non-small cell lung cancer; VN: vessel normalization; DFS: disease-free survival; OS: overall survival; DCE-MRI: dynamic contrast enhance magnetic resonance imaging; MTT: mean transient time; VNI: vascular normalization index; 5-FU: 5-fluorouracil.

The first studies investigating the influence of the VEGF neutralizing antibody bevacizumab on tumor vessel normalization were performed in rectal cancer patients. Bevacizumab monotherapy decreased the interstitial fluid pressure, while tumor blood vessels were covered with more pericytes as compared to pre-therapy investigations [67]. Long-term follow-up of the subsequent phase II study showed high local tumor control rate and promising disease-free and overall survival upon neoadjuvant bevacizumab treatment in combination with standard chemoradiotherapy [60], suggesting that vessel normalization features might induce a better therapeutic outcome. In support of this hypothesis, treatment with cediranib (a VEGFR tyrosine kinase inhibitor) resulted in increased tumor perfusion and prolonged survival in a subgroup of patients with recurrent glioblastoma multiforme [61]. Accordingly, improved tumor vascularization after bevacizumab combination therapy with carboplatin and nab-paclitaxel was associated with longer survival in patients with advanced non-small cell lung cancer [62]. Overall, the abovementioned studies may imply that antiangiogenic therapy regimens that promote tumor vessel normalization offer benefit to cancer patients. Clearly, the number of clinical studies showing beneficial outcome of tumor vessel normalization is still limited, and additional studies are needed to corroborate these initial clinical findings. In addition, it is of utmost importance to identify predictive biomarkers for vascular normalization and therapy stratification in future clinical trials [19].

A recent clinical trial, including a small number of patients with metastatic renal cell carcinoma treated with bevacizumab in combination with an anti-PD-ligand 1 antibody showed that the combination treatment increases the intra-tumoral infiltration of CD8^+^ T cells, thus enhancing the anticancer immune-specific responses [68]. The ongoing early phase I/II clinical trials will further reveal whether the anti-VEGF therapies in combination with checkpoint inhibitors increase the anticancer effects [69].

Mechanistically, it has been hypothesized (and supported by preclinical evidence) that upon tumor vessel normalization, the improved vessel functionality leads to enhanced tumor delivery of chemotherapeutics [70]. However, some clinical trials investigating the effect of antiangiogenic drugs on tumor delivery showed that antiangiogenic drugs reduced (rather than increased) the delivery of chemotherapy or biological drugs [71,66]. A reduction in tumor perfusion and vessel permeability has been proposed to explain the decreased tumor uptake of chemotherapeutics upon bevacizumab therapy [72]. Further, drug penetration in tumors was enhanced only when the chemotherapeutic agent was administered within a defined interval after anti-VEGF therapy [73,74]. Together, while tumor vessel normalization is an attractive strategy to impede metastasis and improve chemo-, radio-, and immunotherapy in preclinical models, and initial signs have been recognized in clinical settings, other studies also highlight the importance of testing different treatment regimen schedules and dosing of bevacizumab in order to examine their effect on perfusion and permeability and to identify the normalization window in which the antiangiogenic agent exerts beneficial effects. Nonetheless, it will remain challenging to clinically translate the preclinical efficacy of VEGF-signaling blockers to induce long-lasting tumor vessel normalization required for optimal therapeutic benefit. Hence, we will need additional agents with a completely different mechanism, which are capable of inducing persistent tumor vessel normalization (as for instance documented in mice for chloroquine, PHD2 gene haplodeficiency, etc. – see above).

### 2.6. Tumor vessel normalization by targeting EC metabolism

An entirely different and new antiangiogenic approach to promote tumor vessel normalization is to target EC metabolism. This strategy is based on the postulate that EC metabolism is the engine onto which pro-angiogenic signals like VEGF and others converge and that ‘cooling down the overheated metabolism’ of ECs can paralyze angiogenic ECs and reduce pathological angiogenesis, regardless of how many angiogenic signals are still present upon neutralization of VEGF [3,75,76]. Since ECs are highly glycolytic [77], targeting glycolysis might provide an alternative new therapeutic opportunity for reducing pathological angiogenesis. Indeed, 6-phosphofructo-2-kinase/fructose-2,6-bisphosphatase 3 (PFKFB3) is a key regulator of glycolysis in ECs. Inhibition of PFKFB3, either by genetic loss in ECs or by global pharmacological blockade with the small molecule 3PO (3-(3-pyridinyl)-1-(4-pyridinyl)-2-propen-1-one), reduces vessel sprouting by inhibiting EC proliferation and migration, not only in physiological but also in pathological conditions of inflammation and tissue injury [77,78]. Notably, however, PFKFB3 silencing or blockade reduces EC glycolysis only partially by no more than 35%, but still sufficient to normalize the hyper-glycolysis of sprouting ECs to maintenance levels found in quiescent ECs [77,78]. Thus, even a modest decrease in glycolysis was sufficient to impair vessel sprouting by promoting quiescence [78]. In addition, the effect of 3PO *in vivo* was transient because of its short half-life (30 min) and rapid clearance. As a result of the partial, transient reduction (not elimination) of glycolysis, and the fact that ECs are more glycolysis addicted than other cell types, the effect of 3PO was well tolerated [78,79].

This type of anti-glycolytic approach differs substantially from previous anti-glycolytic anticancer therapies, which were not always successful [80,81], mainly because they attempted to eliminate glycolysis completely and permanently, which causes adverse effects. Indeed, the non-metabolizable glucose analog 2-deoxy-d-glucose (2DG), which reduces glycolysis by 80%, causes ATP depletion and EC death [78]. Since high 2DG doses are needed to compete with the high levels of glucose in the blood, its effects are toxic.

**Figure 2.**
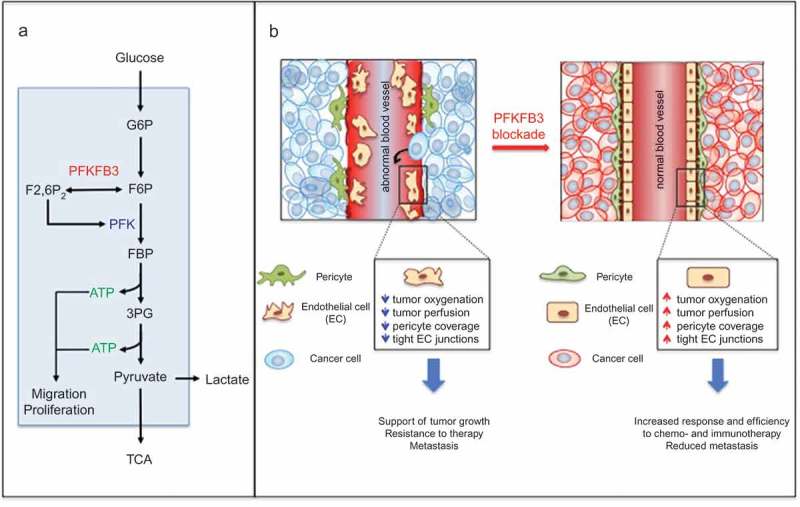
Targeting endothelial cell metabolism induces tumor vessel normalization. a. Schematic representation of the glycolytic pathway converting glucose into pyruvate. PFKFB3 is a key regulator of glycolysis by producing fructose-2,6-bisphosphate (F2,6P_2_), the most potent allosteric activator of phosphofructokinase-1 (PFK-1). G6P, glucose-6-phosphate; F6P, fructose-6-phosphate; F2,6P_2_, fructose-2,6-bisphosphate; PFK, phospho-fructokinase; 3PG, 3-phospho glyceraldehyde; TCA, tricarboxylic acid cycle; ATP, cellular adenosine 5ʹ-trisphosphate.b. Upon inhibition of PFKFB3 in hyperglycolytic tumor endothelial cells, tumor vessels show smoother endothelial surface, reduced intercellular gaps, more prominent basement membrane and increased pericyte coverage. All these changes improve tumor vessel perfusion and thereby lower hypoxia, contributing to reduced invasion, intravasation and metastasis. Adapted from [8].

Deletion of both PFKFB3 alleles in ECs decreases angiogenesis and perfusion in tumors [82]. A more recent study showed however that endothelial haplodeficiency of PFKFB3 does not inhibit tumor growth but reduces metastasis and improves the delivery and response to chemotherapy, by normalizing tumor vessels [83]. Treatment with a low dose of 3PO, which reduces glycolysis by only 15% in ECs, induces similar effects [83]. Since ECs’ lining tumor blood vessels have a much higher glycolytic rate than healthy ECs, they are more sensitive to PFKFB3 blockade [83]. This can explain why even the deletion of one allele of PFKFB3 or the use of a low dose of 3PO already induces tumor vessel normalization [83]. Unlike traditional antiangiogenic agents [84–86], PFKFB3 haplodeficiency or blockade does not reduce tumor vessel density or total vascular area. Instead, PFKFB3 inhibition enlarges the vessel lumen and stabilizes tumor vessels by increasing vessel maturation through pericyte recruitment. These morphological changes improve tumor perfusion and thereby lower tumor hypoxia [83]. Overall, PFKFB3 blockade reduces metastasis and increases chemotherapy delivery and efficacy (Figure 2).

## 3. Conclusions

Since the concept of tumor vessel normalization for anticancer therapy was proposed in 2001, promising progress has been made in preclinical studies and clinical trials. By restoring the balance between pro- and antiangiogenic factors in the tumor microenvironment, antiangiogenic therapy using adequate antiangiogenic agents and delivery regimens is able to ‘heal’ the perturbed tumor vessels and to restore a more normal tumor vasculature, capable of more efficiently delivery of cytotoxic drugs and other therapies such as immunotherapies to tumors. While preclinical studies have amply demonstrated the therapeutic benefit of tumor vessel normalization strategies, translation to the clinic is now required to provide proof of evidence for similar benefit in cancer patients. However, this requires clinical development of new alternative strategies, capable of inducing more long-lasting and more efficient tumor vessel normalization. Targeting endothelial metabolism by lowering glycolysis in tumor ECs is emerging as a novel anticancer therapeutic approach, capable of inducing tumor vessel normalization, and hence reducing metastasis while improving chemo-, radio-, and immunotherapy.

## 4. Expert opinion

More than a decade of clinical experience reveals that we have yet to realize the full potential of antiangiogenic therapy. Most antiangiogenic agents received market authorization after providing proof of efficacy in a phase III trial that PFS or overall survival is prolonged compared to the most efficacious available therapy. However, these studies did not sufficiently investigate the fundamental mechanisms of how antiangiogenic drugs in combination with chemotherapy produce clinical benefit, and the exact time window in which the antiangiogenic agents exert the greatest benefit remains largely undefined.

Cancer patients receive disrupting scheduling of anti-VEGF regimens, which are often interrupted because of toxicity, resistance, or high costs. Discontinuation of antiangiogenic therapy might cause a rebound effect, leading to disease progression and metastasis (as observed at least in preclinical studies [11,12,85,87]). One possible alternative, which has been poorly explored to date, might be to administer low (i.e. lower than maximally tolerated) doses of antiangiogenic drugs with the objective to promote tumor vessel normalization. In this case, the increased anticancer activity would not result from vessel pruning upon administration of high doses of antiangiogenic drugs, but rather from the restoration of a more functional tumor microenvironment that not only facilitates the delivery of chemotherapy and immune effector cells to the tumor but also impairs metastasis. Since targeting VEGF signaling often normalizes tumor vessels transiently and may ultimately provoke vessel regression, the development of alternative antiangiogenic strategies with a fundamentally distinct mechanism is mandated.

The recent data that the glycolytic regulator PFKFB3 controls vessel sprouting and its blockade promotes tumor vessel normalization [83] raise the question if strategies targeting EC metabolism could increase the response of cancer patients to current anticancer treatment and represent a complementary or alternative antiangiogenic approach. A clinical trial using a small chemical PFKFB3 blocker has been initiated to target, in the first instance, cancer cells by using maximally tolerated doses [88], based on preclinical studies designed to inhibit cancer cell proliferation [89]. Since ECs are very sensitive to even small changes in glycolysis levels and, in fact, more responsive to PFKFB3 blockade than various cancer cell lines *in vitro* [83], a clinical trial with another design using lower doses of the PFKFB3 blocker will be required to test whether PFKFB3 blockade can impair metastasis, while improving standard care therapy by promoting tumor vessel normalization. It will remain to be determined whether a high, or rather a low, dose of the PFKFB3 blocker is capable of inducing tumor vessel normalization, and its associated therapeutic benefits of reduced metastasis and improved response to chemotherapy.

## Article highlights

- Anti-VEGF therapy promotes vascular pruning
- Excessive vascular pruning increases the risk of metastasis and impairs drug delivery and efficacy
- Tumor vessel normalization represents an alternative strategy for antiangiogenic cancer treatment
- Targeting endothelial cell glycolysis normalizes tumor vessels
- PFKFB3 blockade reduces metastasis and promotes chemotherapy efficacy

This box summarizes key points contained in the article.
